# Chemical Markers to Distinguish the Homo- and Heterozygous Bitter Genotype in Sweet Almond Kernels

**DOI:** 10.3390/foods9060747

**Published:** 2020-06-05

**Authors:** Stefania Vichi, Morgana N. Mayer, Maria G. León-Cárdenas, Beatriz Quintanilla-Casas, Alba Tres, Francesc Guardiola, Ignasi Batlle, Agustí Romero

**Affiliations:** 1 Departament de Nutrició, Ciències de l’Alimentació i Gastronomia, Campus De l’Alimentació de Torribera, Facultat de Farmàcia i Ciències de l’Alimentació, Universitat de Barcelona, 08921 Santa Coloma de Gramenet, Spain; mgleonc@hotmail.com (M.G.L.-C.); beatrizquintanilla@ub.edu (B.Q.-C.); atres@ub.edu (A.T.); fguardiola@ub.edu (F.G.); 2 Institut de Recerca en Nutrició i Seguretat Alimentària (INSA-UB), Universitat de Barcelona (UB), 08921 Santa Coloma de Gramenet, Spain; 3 Instituto de Biología Agrícola de Mendoza (IBAM-CONICET), Facultad de Ciencias Agrarias, Universidad Nacional de Cuyo, 5505 Mendoza, Argentina; mmayer@mendoza-conicet.gob.ar; 4 Institute of Agrifood Research and Technology (IRTA)-Mas Bové Ctra. Reus-El Morell km 3.8, 43120 Constanti, Spain; Ignasi.Batlle@irta.cat (I.B.); Agusti.Romero@irta.cat (A.R.)

**Keywords:** *Prunus dulcis*, *Prunus amygdalus*, breeding, almond kernel, bitterness, genotype, benzaldehyde, chemical marker

## Abstract

Bitterness in almonds is controlled by a single gene (*Sk* dominant for sweet kernel, *sk* recessive for bitter kernel) and the proportions of the offspring genotypes (*SkSk*, *Sksk*, *sksk*) depend on the progenitors’ genotype. Currently, the latter is deduced after crossing by recording the phenotype of their descendants through kernel tasting. Chemical markers to early identify parental genotypes related to bitter traits can significantly enhance the efficiency of almond breeding programs. On this basis, volatile metabolites related to almond bitterness were investigated by Solid Phase Microextraction-Gas Chromatography-Mass Spectrometry coupled to univariate and multivariate statistics on 244 homo- and heterozygous samples from 42 different cultivars. This study evidenced the association between sweet almonds’ genotype and some volatile metabolites, in particular benzaldehyde, and provided for the first time chemical markers to discriminate between homo- and heterozygous sweet almond genotypes. Furthermore, a multivariate approach based on independent variables was developed to increase the reliability of almond classification. The Partial Least Square-Discriminant Analysis classification model built with selected volatile metabolites that showed discrimination capacity allowed a 98.0% correct classification. The metabolites identified, in particular benzaldehyde, become suitable markers for the early genotype identification in almonds, while a DNA molecular marker is not yet available.

## 1. Introduction

Almond (*Prunus dulcis* (Mill.), D. A. Webb; syn. *P. amygdalus*, Batsch.) is the main nut tree worldwide and almonds have an important commercial value, with an annual world production exceeding 3,000,000 tons in shell [1]. Sweet almond kernels are widely consumed raw or minimally processed, as well as used as an ingredient in food products. Genetic improvement programs for almonds in different countries such as Spain, Australia and the United States, have been selecting and releasing cultivars with the best agronomic and industrial characteristics [2,3,4,5,6]. One of the important aspects in the manufacturing of almond products is bitterness, since the presence of bitter almonds in sweet almond batches is detrimental to the quality of the final product. Bitterness in almonds kernels is due to the presence of the cyanogenic glucoside amygdalin, which undergoes enzymatic hydrolysis by β-glucosidases upon disruption of tissues, to form glucose, hydrogen cyanide and benzaldehyde [7]. This enzymatic breakdown and the concomitant liberation of hydrogen cyanide and benzaldehyde are responsible for the marzipan-like and bitter taste of some kernels [8,9,10]. The precursor prunasin is produced in plant mother tissues and translocated into the developing kernel, where it is transformed in amygdalin [7]. Thus, the genotype of the mother plant controls kernel bitterness, which is the same for all the kernels of a tree [11,12,13].

A single gene controls the bitter character in almond with a sweet allele (*Sweet Kernel*, *Sk*) that is dominant over the bitter one (*sk*) [14,15,16]. The gene *Sk* has been mapped in linkage group five of the almond genome [17] and its chromosome 5 position and function were recently revealed [18,19]. After crossing, three possible genotypes are expected: homozygous *SkSk* (sweet), *sksk* (bitter), and heterozygous *Sksk* (sweet or semi-bitter). There is no genetic distinction between sweet and semi-bitter cultivars, but Dicenta and García [12] suggested that semi-bitter forms correspond to heterozygous trees (*Sksk*) in which the recessive allele may induce some slightly bitter taste. All the semi-bitter forms are heterozygous, but not all the heterozygous forms are semi-bitter.

Almond is an outcrossing species, mostly self-incompatible, that has been made self-compatible through domestication and breeding. Commercially, there are orchards of self-incompatible cultivars in USA and Australia and self-compatible (self-fertile) cultivars mainly in the Mediterranean region. Usually, cross- or self-pollination, respectively, is favored using beehives in the orchards, which are open-pollinated. In almond scion breeding programs, many cultivars used as progenitors are heterozygous, and homozygous bitter progenitors can sometimes be advantageous to introduce some favorable agronomic traits in the progeny [16]. After crossing, the ratio of each genotype (*SkSk, Sksk, sksk*) in the offspring depends on the progenitors’ genotype. When one of the progenitors has a dominant homozygous genotype (*SkSk*) the entire progeny shows a sweet phenotype, but the descendants with bitter phenotype are around 25% when crossing two heterozygous cultivars and around 50% when crossing heterozygous with recessive homozygous ones [12]. As seedlings with bitter phenotype must be discarded during the selection process, the efficiency of the genetic improvement programs can be significantly enhanced by reducing the crossing of heterozygous individuals among them. With this scope, a classification of the genitor cultivars into homozygous or heterozygous for the sweet character is necessary. At present, the genotype of almond cultivars and selections is deduced after crossing by recording the phenotype of their descendants through kernel tasting, and quantifying the seedlings with sweet and bitter kernel [12,20], because molecular markers are not well developed yet to be useful [17,21]. This implies a long waiting time until cropping (3–4 years). All semi-bitter descendants can be classified as heterozygous (*Sksk*) according to Dicenta and García [12], but their differentiation from sweet ones is difficult and requires a trained sensory panel, and this criterion would not consider the rest of heterozygous cases presenting a completely sweet kernel.

Some efforts have been made to find chemical markers for early genotype identification in almond cultivars and selections used in breeding programs. With this aim, the content of amygdalin in almond kernels has been monitored as a function of the phenotype and genotype of several almond cultivars [22,23]. Although a clear difference was evidenced in the content of amygdalin between sweet and bitter almond kernels, a high variability was observed within the sweet phenotype. In fact, amygdalin in bitter cultivars ranged from 2000 to 60,000 mg/kg, while in semi-bitter and sweet cultivars it ranged from 20 to 1772 mg/kg and from not detectable (n.d.) to more than 200 mg/kg, respectively [9,24], thus presenting overlapping ranges of amygdalin concentration. In particular, the amygdalin content did not allow a clear distinction between sweet kernelled heterozygotes (*Sksk*) and sweet kernelled homozygotes (*SkSk*), in which it fluctuated from 18.7 to 80.2 mg/kg and from n.d. to 55 mg/kg, respectively [7,22]. These results suggest that even though amygdalin has a clear correlation with bitterness, this marker is not completely effective in predicting slight differences in bitterness such as those existing between sweet and semi-bitter kernels, and even less effective in detecting possible differences between sweet homo- and heterozygotes. This could be due to the performances of the analytical methods applied for the determination of the cyanogenic glucoside, or to the existence of secondary factors linked to the recessive allele affecting the production of benzaldehyde or other compounds causing bitterness perception.

According to Wirthensohn et al. [10] the overlap of the concentration ranges in sweet and semi-bitter kernel indicates that amygdalin may not be the only compound defining the marzipan-like flavor in sweet almonds. Some authors have pointed out the close correlation between bitter marzipan-like flavor and benzaldehyde, one of the amygdalin catabolites, even at low bitterness intensities assessed in sweet almond cultivars [25]. In addition, other almond volatile compounds, such as benzyl alcohol, revealed higher values in bitter almonds than in sweet almonds [26], and their levels tend to be higher in almonds with higher levels of benzaldehyde [25,27].

On this basis, the concentration of benzaldehyde and other related volatile compounds was monitored in 42 homozygous and heterozygous almond cultivars and selections, with the aim of identifying suitable chemical markers to classify sweet kernel almonds according to their genotype (homozygotes or heterozygotes). With this aim, a Solid Phase Microextraction-Gas Chromatography-Mass Spectrometry (SPME-GC-MS) method was optimized and applied to 244 almond samples obtained from 124 different trees.

## 2. Materials and Methods

### 2.1. Samples

Almonds (*Prunus dulcis* (Mill.), D. A. Webb; syn. *P. amygdalus*, Batsch.) of 41 different cultivars and selections and one feral tree were studied. For 37 of these, their genotypes were previously reported in the literature [12,17,20,28] or determined by IRTA’s almond breeding program. In agreement with these sources, the 42 cultivars and selections consisted of 22 homozygous and 14 heterozygous sweet kernel cultivars, five selections without known genotype and one reference bitter feral tree. Few of these heterozygous cultivars (‘Tuono’, ‘Guara’, ‘Genco’) are described as semi-bitter, although no precise and objective criteria have been set for this classification. Hereinafter, all the samples except the bitter one will be considered as sweet kernel almonds. A total of 244 almond samples were obtained from 124 different trees (Table 1). These samples were produced in 2012 and 2015 in different geographical areas: Constantí and Gandesa in Tarragona and Les Borges Blanques in Lleida (Catalonia, Spain). Out of the 42 cultivars, 10 (8 homozygous and 2 heterozygous) were analysed both in 2012 and 2015. Almonds were collected, shelled and blanched by hand, then packed under vacuum, stored at 2–8 °C, and analysed within three months.

### 2.2. Chemical Reagents

4-Methyl-2-pentanol, ethyl acetate, hexanal, 1-Penten-3-ol, 3-Methylbutan-1-ol, 1-Hexanol, nonanal, 1-Heptanol, benzaldehyde, phenylethyl alcohol and benzyl alcohol were from Sigma-Aldrich Co (St. Louis, MO, USA). Ultrapure water (Milli-Q Millipore Corporation, Billerica, MA, USA) was used.

### 2.3. Sample Preparation and Solid Phase Microextraction (SPME) Conditions

The SPME fiber divinylbenzene/carboxen/polydimethylsiloxane fiber (50/30 µm, 2 cm long from Supelco Ltd., Bellefonte, PA, USA) was selected as being the most suitable for compounds with a wide range of molecular weight and polarity. The extraction of volatiles was performed on a suspension of ground almonds in aqueous solution on the basis of preliminary results obtained by comparing the uptake of volatiles obtained from ground almonds (1 g) and from ground almonds in aqueous suspension (1 g in 2 mL of ultrapure water). A multilevel factorial experiment was then applied to optimize the rest of the parameters affecting the extraction of volatile compounds: extraction temperature (40, 50, 60 °C), extraction time (20, 30, 40 min), sample amount (1, 1.5 g) and pH of the suspension (3.5, 7). The optimized factorial design consisted of 20 experiments performed in duplicate and randomized (Supplementary Table S1). The dependent variables were the GC-MS responses of 12 representative compounds of the volatile profile, belonging to different chemical families (Table 2). The influence of the different factors was evaluated by means of a normalized Pareto diagram, elaborated with the chromatographic responses of each analyte in the different extraction conditions. The optimal value of each factor involved in the extraction was statistically calculated and the best extraction conditions were chosen for the analysis.

Finally, almond samples were analysed as follows: 10 g of skinless almonds were ground during 1 min using a domestic grinder (Iberica Group, Barcelona, Spain), then 1 g of the sample was suspended in 2 mL of ultrapure water (pH 7) in a 10 mL vial. The sample was spiked with 4-methyl-2-pentanol (Sigma-Aldrich, St. Louis, MO, USA) to a final concentration of 0.5 μg/g of almonds and sealed with a PTFE-silicone septum. The vial was placed in a water bath at 60 °C under magnetic stirring, and the SPME fiber was maintained for 40 min in the sample headspace. The volatile compounds of the fiber were desorbed for 1 min at 260 °C in the gas chromatograph injection port.

Intra-day repeatability was assessed by analyzing the same almond sample five times and calculating the percent relative standard deviation (Supplementary Table S2).

### 2.4. Gas Chromatography-Mass Spectrometry (GC-MS) Analysis

GC-MS analyses were performed in 2012 on a Thermo Scientific Trace GC Ultra coupled to a quadrupole mass selective spectrometer DSQ II (Thermo Scientific, Bremen, Germany) and in 2015 on an Agilent GC 6890N coupled to a quadrupole mass selective spectrometer 5973 (Agilent Technology, Palo Alto, CA, USA). Both were equipped with a split-splitless injection port. Helium was the gas carrier, at linear velocity of 1 mL/min. The separation of the volatiles was performed by a column Supelcowax-10 (30 m × 0.25 mm i.d., 0.25 μm film thickness), purchased from Supelco Ltd (Bellefonte, PA, USA). The temperature of the column was held at 40 °C for 5 min and increased to 250 °C at 6 °C/min. Electron impact mass spectra were recorded at 70 eV ionization energy in the 35–250 *m/z* range, 2 scan/s.

Volatile compounds were identified by comparison of their mass spectra and retention times with those of standard compounds or tentatively identified by comparing their mass spectra with the reference mass spectra of the Wiley 6.0 library and their linear retention indices with those reported in the literature. For quantitative analysis, relative amounts of volatile compounds were calculated by using the internal standard method. The compounds were quantified by considering the relative response factor to be 1 and were expressed as micrograms per gram equivalents of 4-Methyl-2-pentanol.

### 2.5. Statistical Analysis

Statistical elaboration for the optimization of the SPME conditions was carried out using Statgraphics Plus 5.1© (Statgraphic Technologies Inc., The Plains, VA, USA). Four factors were tested at three or two levels, as previously described. The factorial design consisted of 20 experiments performed in duplicate. The normalized results of the experimental design, evaluated at a significance level of 5%, were analysed using a standardized Pareto diagram, which shows a frequency histogram where the length of each bar in the graph is proportional to the absolute value of its standardized effect. The significance of the factors studied and the optimal values for each factor were established by means of an ANOVA and a regression analysis of the model, respectively. The results were considered significant with values of *p* < 0.05.

Univariate statistical analysis was performed with SPSS software v25© (IBM Corp., NY, USA). Student’s t-test was applied to compare homo- and heterozygous groups, and bilateral Pearson correlations were assessed between benzaldehyde and the compounds presenting significant differences by the t-test, and between benzaldehyde and bitterness. In all cases, *p* < 0.05 was considered significant. Analysis of variance by General Linear Model (GLM) of SPSS was carried out according to the harvest year and geographical production area.

Multivariate analysis was carried out with SIMCA software v13.0© (Umetrics AB, Sweden). With the variables selected by univariate statistics (6 variables) and after data pre-processing (scaling to unit variance), a Principal Component Analysis (PCA) was developed to explore the natural clustering of samples and detect potential outliers (according to Hotelling’s T2 range and distance to the model parameters). A Partial Least Square-Discriminant Analysis (PLS-DA) classification model was then built with the same variables to classify the samples into homo- or heterozygous categories.

## 3. Results and Discussion

In almonds, individuals with sweet kernel phenotype can present homozygous (*SkSk*) or heterozygous (*Sksk*) genotype. To classify them according to this genotype, suitable metabolic markers were investigated after optimizing a proper analytical method.

### 3.1. Optimization of SPME-GC-MS Method for the Assessment of Volatile Compounds

A 29% increase in total chromatographic area was observed by analyzing ground almonds in suspension in comparison to dry extraction (Supplementary Table S3). This greater efficiency is justified by a better mass transfer due to a greater exposure of the surface of the almond particles compared to direct extraction, in which these particles tend to agglomerate. The presence of water could also favor enzymatic reactions leading to some volatiles related to almond bitterness [26].

Table 2 shows the optimal values for the extraction variables that were found to significantly influence the extraction of each volatile compound. The temperature and the extraction time were the parameters with the highest influence on volatiles uptake. As expected, for most compounds an increased chromatographic response was observed at 60 °C and 40 min. The compounds whose uptake was significantly influenced by pH showed a better extraction at pH 7. The amount of sample only showed a significant effect on few volatile compounds, and it was maintained at 1 g to favor a proper stirring during the extraction.

### 3.2. Univariate Statistical Analysis of Raw Almond Volatile Components

Thirty compounds were detected in the headspace of the samples under study (Supplementary Table S2), most of which were previously described in almonds [29,30]. To identify metabolites whose biogenesis could be related to the almond genotype (*SkSk, Sksk*), we focused on the compounds that presented significant differences between homo- and heterozygous almonds when assessed by univariate analysis (Table 3). Benzaldehyde, benzyl alcohol and 1-penten-3-ol presented significantly higher concentrations in kernels from heterozygous (*Sksk*) cultivars, while branched aldehydes 2- and 3-methylbutanal, and branched alcohols 2-Methylpropan-1-ol, 3-Methylbutan-1-ol, 3-Methyl-3-buten-1-ol and 3-Methyl-2-buten-1-ol were more abundant in homozygous (*SkSk*) ones. A relationship with the recessive allele could be hypothesized for those of them that presented clear trends according to the genotype: *SkSk*<*Sksk*<*sksk*, such as benzaldehyde and benzyl alcohol; or *SkSk*>*Sksk*>*sksk*, such as branched alcohols 2-Methylpropan-1-ol, 3-Methylbutan-1-ol, 3-Methyl-3-buten-1-ol and 3-Methyl-2-buten-1-ol (Table 3). All these compounds’ results significantly correlated with benzaldehyde in all the sweet almond phenotypes (Table 3). On the contrary, branched aldehydes and 1-Penten-3-ol did not follow any of these trends, and they did not significantly correlate with benzaldehyde, suggesting that their formation could be driven by varietal factors unrelated to the kernel bitterness. For this reason, they were not further considered as possible genotype markers in sweet almonds. Although the harvest year and the production area influenced the concentration of the selected volatiles (Supplementary Table S4), the differences between *SkSk* and *Sksk* groups were high enough to allow the differentiation of these genotypes in spite of the annual and geographical variability.

While bitterness and marzipan-like flavor had been previously related to benzaldehyde and benzyl alcohol in semi-bitter and bitter almonds [10,25,31], no data were available about the occurrence of these compounds in sweet almonds according to their genotype. While benzaldehyde is known to proceed from amygdalin catabolism [7,8,9], the biosynthesis of benzyl alcohol in almonds has not been elucidated. Kwak et al. [26] documented that it is formed in bitter almond kernel by enzymatic reactions, which may consist of the reversible enzymatic reduction of benzaldehydes as described in other plants [32]. This would substantiate the association of benzyl alcohol with benzaldehyde and almonds’ bitter character. In the same way, the enzymatic formation of branched alcohols was predominant in sweet rather than in bitter almond kernels [26], but it was unknown that these compounds were also predominant in homozygous sweet almond genotypes compared to heterozygotes.

**Figure 1:**
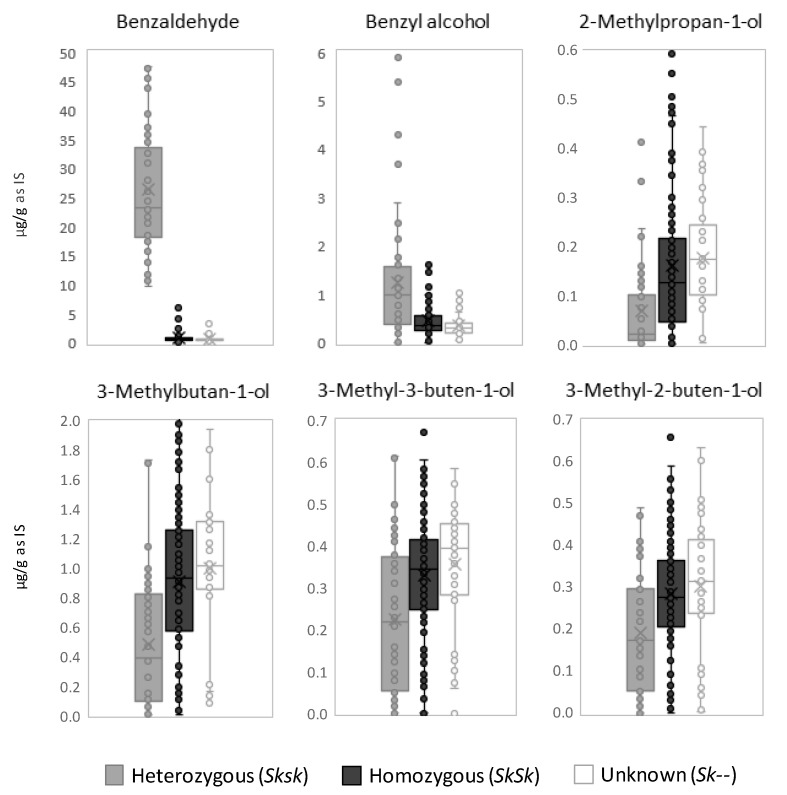
Box-and-whisker plots obtained for the selected variables in each group of sweet almond samples: heterozygous (*Sksk*), homozygous (*Sksk*) and samples with unknown (*Sk--*) genotype.

Box-and-whisker plots were built to explore the concentration ranges of the selected compounds and their capacity to differentiate homo- and heterozygous sweet genotypes (Figure 1). While most of the compounds presented certain overlap in the ranges of homo- and heterozygous groups, benzaldehyde levels allowed a neat distinction between these groups. We report for the first time a discrimination between homo- and heterozygous sweet almond genotypes based on a chemical marker, which resulted from the analysis of more than 200 samples from 36 distinct cultivars. These results indicate that benzaldehyde performed better than reported for amygdalin to differentiate homo- and heterozygous sweet almond kernels [7,22]. This could be the consequence of a higher sensitivity in the detection of benzaldehyde, which led to differentiation even between kernels of very low bitterness. This was sustained by the significant correlation (Pearson correlation = 0.787, *p* < 0.001) between benzaldehyde and the mean bitterness intensity of the sweet cultivars under study, assessed by IRTA’s almond sensory panel on samples from previous harvest years (Table 1). In addition, we could hypothesize that the accumulation of amygdalin in the kernel is not the only effect of the recessive bitter allele in heterozygotes, and that the latter could influence other enzymatic reactions such as the catabolic routes yielding benzaldehyde and related compounds, as well as the synthesis of branched alcohols.

Benzaldehyde could represent a suitable chemical marker for the early genotype identification in almond cultivars and selections used in breeding programs. In this regard, samples from the five sweet almond selections without known genotype (IRTA-7, IRTA-11, ‘Cambra’, ‘Felisia’ and ‘Soleta’) were classified as homozygous cultivars (Figure 1). This classification may be verified once the bitter character segregation data are available in the progeny of these cultivars.

Although the homo- and heterozygous sweet almonds considered in the present study could be discriminated directly by their levels of benzaldehyde, all the metabolites whose biogenesis seemed to be linked to the almond genotype could be useful to support this classification as confirmation parameters or in multivariate models.

### 3.3. Multivariate Statistical Analysis of Raw Almond Volatile Components

**Figure 2:**
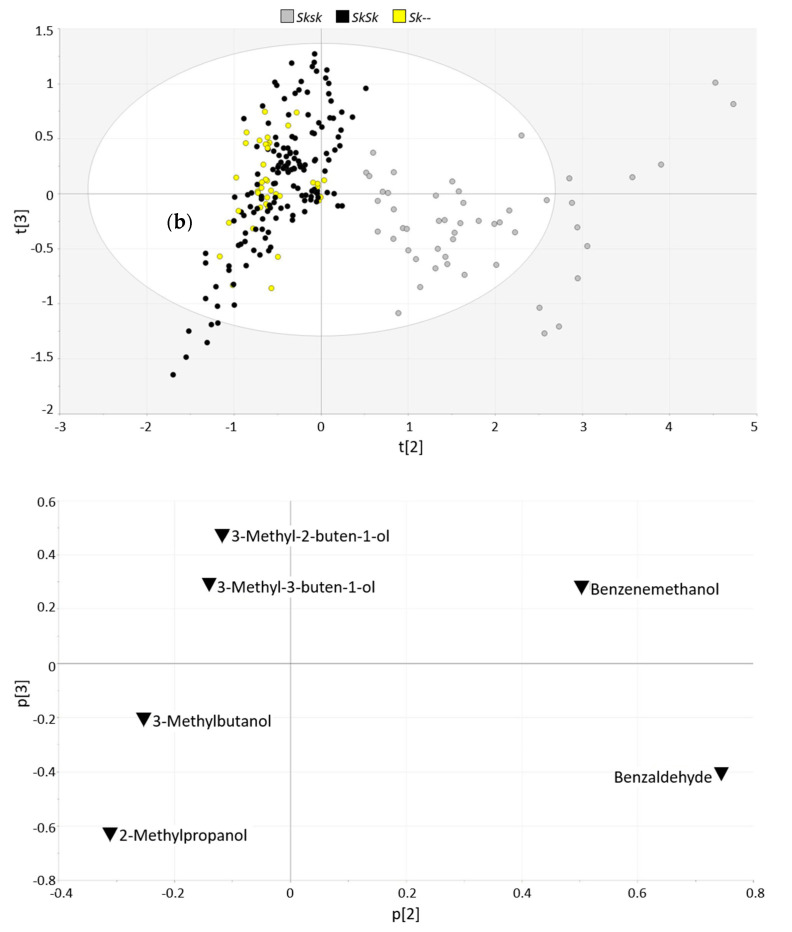
Principal Component Analysis (PCA) (*n* = 242, 6 variables, scaled to unit variance, 3 PC, 94.7% total variance explained). (**a**) Scores plot and (**b**) loadings plot corresponding to PC2 and PC3 (19.6% and 4.8% total variance explained, respectively).

A multivariate statistical approach based on various potential genotype markers was carried out to support the differentiation allowed by benzaldehyde with the aim of providing a more reliable classification tool. PCA was carried out with the biomarkers previously selected by univariate analysis (3 Principal Components (PCs) accounted for 94.7% of the total variance explained, no outliers detected). While PC1 seemed to depend on varietal characteristics not linked to the bitter allele (data not shown), the scores and loadings plots corresponding to PC2 and PC3 confirmed that a clear differentiation between hetero- and homozygous individuals (Figure 2a) was driven by benzaldehyde and benzyl alcohol, and branched alcohols, respectively (Figure 2b). PC2 was the component that mainly contributed to the differentiation between hetero- and homozygous individuals (19.6% of explained variance). As expected, benzaldehyde was the variable that mainly contributed positively to this component, followed by benzyl alcohol (PC2 loadings 0.744 and 0.503, respectively), while 2-methyl propanol, 3-Methylbutan-ol, 3-Methyl-3-buten-1-ol and 3-Methyl-2-buten-1-ol were the ones mainly contributing negatively to this PC (PC2 loadings −0.310, −0.252, −0.140 and −0.113, respectively).

**Figure 3:**
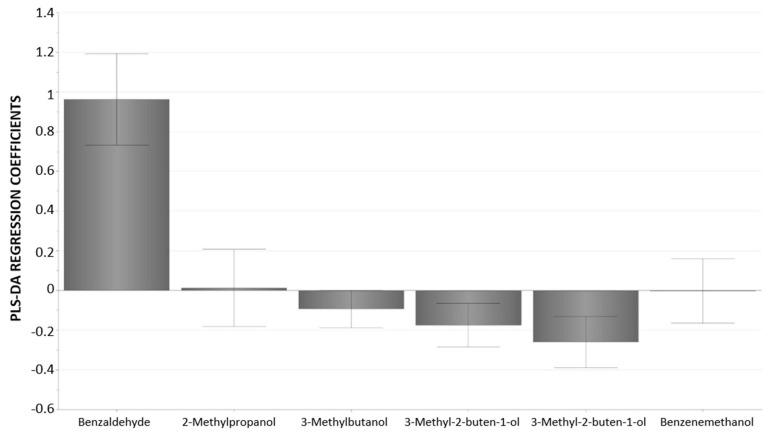
Partial Least Square-Discriminant Analysis (PLS-DA) regression coefficients for the heterozygous (*Sksk*) category, with confidence intervals derived from jack-knifing.

On this basis, to dispose of a classification tool for sweet almonds based on all volatile compounds whose biogenesis seemed to be linked to their genotype, a supervised discriminant technique was applied to find the maximum correlation between the data and each of the categories of interest (heterozygous vs homozygous). A PLS-DA classification model developed according to the almond genotype and based on the previously selected variables provided a 98.0% correct classification, as obtained by leave-10%-out cross-validation (Table 4). The corresponding predicted values are reported in Supplementary Table S5. The permutation test (*n* = 20) indicated that the model was not over-fitted according to the Q^2^ scores (Model’s Q^2^ = 0.81, permutation models’ Q^2^ < 0). Moreover, PLS-DA regression coefficients confirmed the major role of benzaldehyde in the classification model and evidenced the lower but significant contribution of some branched alcohols (Figure 3).

Four heterozygous samples out of 203 were misclassified by the PLS-DA, three ‘Nonpareil’ and one ‘FGFP092’. Other samples from these cultivars were correctly classified by the model. All the samples from these cultivars could be well distinguished from homozygous samples by considering only the benzaldehyde content (Figure 1). The slight reduction in the classification efficiency observed by PLS-DA was compensated by a higher classification reliability, given by the application of an approach based on various independent variables.

According to the PLS-DA model, and in agreement with the benzaldehyde content, all the samples belonging to the five cultivars with unknown genotype were classified as homozygous, according to their predicted values (Supplementary Table S6). Such classification was feasible according to their genealogy.

## 4. Conclusions

In conclusion, the results obtained in this work evidenced the association between sweet almonds’ genotype and some volatile metabolites and provided for the first time chemical markers to discriminate between homo- and heterozygous sweet almonds. In particular, the amount of benzaldehyde, assessed by a simple, rapid, automatable and affordable technique such as SPME-GC-MS allowed to differentiate between the homo- and heterozygous samples analyzed in the study (*n* = 203) and to tentatively classify almond kernels with unknown genotype (*n* = 39). Moreover, the PLS-DA classification model built with selected independent metabolites that had discrimination capacity and were thus more likely to provide a greater reliability to the classification, allowed 98.0% of correct category assignment. The selected metabolites, and in particular benzaldehyde, represent suitable chemical markers for the early genotype identification in almond cultivars and selections used in breeding programs. While a DNA molecular marker is not available, this technique can be used to distinguish homo- and heterozygous bitter genotypes in sweet almond and thus it is useful both to determine genotypes of parents for further breeding or screening unwanted seedlings derived from crosses when breeding.

## Figures and Tables

**Table 1:** Almond samples’ pedigrees, harvesting year, tree and sample number, *Sk* genotype and bitterness.

	Cultivar/Selection	2012 (*n*)		2015 (*n*)		Genotype ^a^			Ref.	Bitterness ^b^(0–10)
		Trees	Samples	Trees	Samples					
1	IRTA-7 (Lauranne × OP ^c^)	8	17			unknown			^d^	na ^e^
2	IRTA-9 (Masbovera × Lauranne)	2	4			*SkSk*			^d^	na
3	IRTA-4 (A-202 × FGFP092)	3	5	1	2	*SkSk*			^d^	na
4	IRTA-10 (4-665 × Lauranne)	2	4			*SkSk*			^d^	0
5	IRTA-12 (4-665 × Lauranne)	3	6			*SkSk*			^d^	na
6	IRTA-11 (Primorskyi × Cristomorto) × IRTA-7)	2	4			unknown			^d^	na
7	IRTA-8 (Anxaneta × IRTA-4)	2	4			*SkSk*			^d^	0.7
8	Belona (Blanquerna × Belle d’Aurons)	2	4			*SkSk*			^d^	na
9	Cambra (Ferragnes × Tuono ^f^)	2	4			unknown				na
10	Constantí (FGFD2 × OP)	7	14			*SkSk*			^d^	na
11	Desmayo Largueta (Spanish local)	2	4				*Sksk*		[20]	1.8
12	Felisia (Titan × Tuono)	2	4			unknown			^d^	na
13	Ferragnes (Cristomorto × Aï)	2	4	1	2	*SkSk*			[12,20]	0.5
14	Francolí (Cristomorto × Tuono)	7	13	1	2	*SkSk*			[20]	0
15	Glorieta (Primorskiy × Cristomorto)	6	14	1	2	*SkSk*			[20]	0
16	Guara (syn. Tuono)	6	11	1	2		*Sksk*		[28]	2.8
17	Lauranne (Ferragnes × Tuono)	2	4			*SkSk*			[20]	0.3
18	Marcona (Spanish local)	2	4	1	2		*Sksk*		[20]	0.3
19	Marinada (Lauranne × Glorieta)	7	13	1	2	*SkSk*			^d^	0.2
20	Marta (Ferragnes × Tuono)	3	5				*Sksk*		[17]	na
21	Masbovera (Primorskiy × Cristomorto)	6	11	1	2	*SkSk*			[20]	0.3
22	Nonpareil (Californian reference)	2	4				*Sksk*		[17]	1.1
23	Soleta (Blanquerna × Belle d’Aurons)	5	10			unknown				0.4
24	Tarraco (FLTU18 × Anxaneta)	6	12	1	2	*SkSk*			^d^	0.6
25	Vairo (4-665 × Lauranne)	6	12	1	2	*SkSk*			^d^	0.3
26	IRTA-2 (A-60 × A-192)			1	2	*SkSk*			^d^	0.6
27	IRTA-1 (Wawona × Lauranne)			1	2		*Sksk*		^d^	0.3
28	IRTA-3 (4-665 × Lauranne)			1	1	*SkSk*			^d^	0.6
29	4-665 (Primorskiy × Cristomorto)			1	2	*SkSk*			[20]	0
30	Cristomorto (Italian local)			1	2	*SkSk*			[20]	0.4
31	Falsa Barese (Italian local)			1	2		*Sksk*		[20]	1.3
32	FGFP092 (Ferragnes × Filippo Ceo)			1	2		*Sksk*		[20]	0
33	FGTR13 (Ferragnes × Troito)			1	2		*Sksk*		[20]	2.1
34	FLTU18 (Ferralise × Tuono)			1	2		*Sksk*		[20]	0.3
35	Gabaix (Spanish local)			1	2		*Sksk*		[20]	0.3
36	Garbí (Cristomorto × OP)			1	2	*SkSk*			[20]	0.4
37	Genco (Italian local)			1	2		*Sksk*		[12,20]	3.5
38	Primorskiy (Princess × Nikitskiy)			1	2	*SkSk*			[12,20]	0.3
39	Ramillete (Spanish local)			1	2	*SkSk*			[12,20]	0.4
40	Stelliete (Ferragnes × Tuono)			1	2		*Sksk*		[20]	1.8
41	Tuono ^f^ (Italian local)			1	2		*Sksk*		[12,20]	0.7
42	Bitter almond (Spanish feral)			1	2			*sksk*	^d^	10

^a^: Genotype: *SkSk*, sweet homozygous; *Sksk*, sweet heterozygous; *sksk*, bitter homozygous. ^b^: Bitterness: intensity on a 0–10 sensory scale, assessed by IRTA panel and obtained by averaging data of 1, 2 or 3 harvest years (unpublished data); ^c^: OP, open pollinated; ^d^: IRTA’s breeding records, unpublished; ^e^: na: not available; ^f^: Tuono (syns. Troito, Mazzeto and Guara).

**Table 2:** Results of the factorial design: optimal extraction conditions based on the regression models for the factors that significantly influenced extraction (*p* < 0.05).

RT ^a^ (min)	Compound	T ^b^ (°C)	T ^c^ (min)	pH ^d^	Sample ^e^ (g)
6.36	hexanal	60	40	7	ns ^f^
7.30	2-Methy-1-propanol	40	ns	ns	1
7.98	2-Pentanol	40	ns	ns	ns
10.17	1-Penten-3-ol	ns	40	ns	ns
12.01	3-Methyl-1-butanol	ns	40	ns	1
16.68	2-Methyl-3-buten-1-ol	60	40	7	ns
17.95	1-Hexanol	60	40	7	ns
19.23	nonanal	60	40	ns	ns
21.92	1-Heptanol	60	ns	ns	1.5
24.10	benzaldehyde	60	40	ns	ns
32.69	benzyl alcohol	60	40	ns	ns
33.26	phenylethyl alcohol	60	40	ns	ns

^a^: RT, retention time; ^b^: T, temperature (40; 50; 60 °C); ^c^: t, time (20, 30, 40 min); ^d^: pH, 3.5; 7; ^e^: Sample weight, 1 g; 1.5 g; ^f^: ns, not significant.

**Table 3:** Occurrence of main volatile compounds showing significant differences between genotypes (*SkSk, Sksk*) by Student’s t-test, presented as mean ± standard deviation. Correlation of volatiles with benzaldehyde (in *SkSk, Sksk* samples) are also shown.

Compound	Concentration ^a^			t-Test ^b^	Pearson Correlation ^c^	
	*SkSk* (*n* = 153)	*Sksk* (*n* = 150)	*sksk* (*n* = 2)	*p*	r	*p*
2-Methylbutanal	0.015 ± 0.011	0.007 ± 0.005	0.030 ± 0.000	<0.001	-	-
3-Methylbutanal	0.031 ± 0.019	0.013 ± 0.010	0.037 ± 0.004	<0.001	-	-
2-Methylpropanol	0.16 ± 0.15	0.070 ± 0.089	0.009 ± 0.000	<0.001	−0.236	<0.001
1-Penten-3-ol	0.092 ± 0.098	0.15 ± 0.15	0.011 ± 0.002	<0.001	-	-
3-Methylbutan-1-ol	0.91 ± 0.51	0.50 ± 0.44	0.034 ± 0.008	<0.001	−0.290	<0.001
3-Methyl-3-buten-1-ol	0.33 ± 0.15	0.23 ± 0.17	0.009 ± 0.001	<0.001	−0.213	<0.01
3-Methyl-2-buten-1-ol	0.29 ± 0.14	0.20 ± 0.13	0.012 ± 0.001	<0.001	−0.165	<0.01
benzaldehyde	0.88 ± 1.06	26.3 ± 10.7	129.7 ± 4.7	<0.001	1	-
benzyl alcohol	0.45 ± 0.31	1.29 ± 1.28	33.2 ± 2.5	<0.001	0.767	<0.001

^a^: mean concentration, expressed as μg equivalents of 4-Methyl-2-pentanol (IS)/g of almond; ^b^: significance of the difference between *SkSk* and *Sksk* means as resulted by Student’s t-test; ^c^: bilateral Pearson correlation of compounds with benzaldehyde. Only significant correlations are reported.

**Table 4:** Classification results of the classification model (PLS-DA) developed to discriminate between homo- and heterozygous sweet almond categories (*n* = 203, 6 variables, scaling to unit variance; 3 latent variables), cross-validated by leave 10%-out.

	*n*	Correct Classification	*SkSk*	*Sksk*
*SkSk* (homozygous)	153	100%	153	0
*Sksk* (heterozygous)	50	92%	4	46
Total	203	98.03%	157	46

*n* = 203, Q^2^ = 0.808, RMSEcv = 0.189, ANOVA *p*-value < 0.05.

## Supplementary Materials

The following are available online at https://www.mdpi.com/2304-8158/9/6/747/s1, Table S1: Experiments performed to develop the SPME method, after optimizing the experimental design, Table S2: Volatile compounds identified in the homozygous and heterozygous cultivars and selections under study, Table S3: comparison of chromatographic areas obtained by dry extraction and extraction in aqueous suspension, Table S4: influence of harvest year and production area on the concentration of the selected volatiles obtained by analysis of variance, S5: samples from cultivars with known genotype and their predicted values as the *SkSk* (homozygous) and *Sksk* (heterozygous) class of the PLS-DA model, Table S6: samples from cultivars and selections with unknown genotype, and their predicted values as the *SkSk* (homozygous) class of the PLS-DA model.
