# Supplementary material for: Chemical Markers to Distinguish the Homo- and Heterozygous Bitter Genotype in Sweet Almond Kernels
Source: Foods. 2020 Jun 5;9(6):747. doi: 10.3390/foods9060747 (PMC7353606; doi:10.3390/foods9060747)
Supplement: Supplementary file 1 [file foods-09-00747-s001.docx]

**Table S1.** Experiments performed to develop the SPME method, after optimizing the experimental design, adapting it to a maximum of treatments *n* = 20, performed in duplicate.

| **Experiment** | **Temperature (°C)** | **Time**  **(min)** | **pH** | **Sample Weight (g)** |
| --- | --- | --- | --- | --- |
| 1 | 40 | 20 | 7 | 1 |
| 2 | 40 | 40 | 7 | 1 |
| 3 | 50 | 30 | 7 | 1 |
| 4 | 60 | 20 | 7 | 1 |
| 5 | 60 | 40 | 7 | 1 |
| 6 | 40 | 20 | 3.5 | 1 |
| 7 | 40 | 30 | 3.5 | 1 |
| 8 | 40 | 40 | 3.5 | 1 |
| 9 | 50 | 20 | 3.5 | 1 |
| 10 | 60 | 20 | 3.5 | 1 |
| 11 | 60 | 40 | 3.5 | 1 |
| 12 | 40 | 30 | 7 | 1.5 |
| 13 | 40 | 40 | 7 | 1.5 |
| 14 | 50 | 20 | 7 | 1.5 |
| 15 | 60 | 20 | 7 | 1.5 |
| 16 | 60 | 40 | 7 | 1.5 |
| 17 | 40 | 20 | 3.5 | 1.5 |
| 18 | 40 | 40 | 3.5 | 1.5 |
| 19 | 60 | 20 | 3.5 | 1.5 |
| 20 | 60 | 40 | 3.5 | 1.5 |

**Table S2.** Volatile compounds identified in the homozygous and heterozygous cultivars and selections under study, presented as mean ± standard deviation. Analytical repeatability is also shown

| N | **Compound** | **ID ^a^** | **KI ^b^** | **RSD ^c^ (%)**  **(*n* = 5)** | **Concentration ^d^** | |
| --- | --- | --- | --- | --- | --- | --- |
|  |  |  |  |  | ***SkSk***  **(*n* = 153)** | ***Sksk***  **(*n* = 50)** |
| 1 | acetaldehyde | B,C | 714 | 13 | 0.048 ± 0.035 | 0.041 ±0.028 |
| 2 | dimethylsulfide | B,C | 716 | 23 | 0.004 ± 0.003 | 0.003 ±0.003 |
| 3 | ethylacetate | A | 907 | 12 | 0.035 ± 0.029 | 0.033 ±0.010 |
| 4 | 2-Methylbutanal | B,C | 912 | 11 | 0.015 ± 0.011 | 0.007 ±0.005 |
| 5 | 3-Methylbutanal | B,C | 915 | 9 | 0.031 ±0.019 | 0.013 ±0.010 |
| 6 | ethanol | B,C | 929 | 20 | 0.096±0.108 | 0.058 ±0.065 |
| 7 | pentamethyl heptane | B | 945 | 3 | 2.7 ± 1.8 | 2.3 ± 2.0 |
| 8 | 2-Butanol | B,C | 1027 | n.d. ^e^ | 0.12 ± 0.12 | 0.11 ± 0.14 |
| 9 | methyltridecane | B | 1032 | 8 | 0.12 ± 0.07 | 0.12 ± 0.07 |
| 10 | 1-Propanol | B,C | 1040 | 24 | 0.110 ± 0.054 | 0.084 ± 0.060 |
| 11 | hexanal | A | 1084 | 10 | 0.77 ± 0.51 | 0.84 ± 0.78 |
| 12 | 2-Methylpropan-1-ol | B,C | 1097 | 7 | 0.16 ± 0.15 | 0.070 ± 0.089 |
| 13 | 2-Pentanol | B,C | 1123 | 16 | 0.079 ± 0.051 | 0.062 ± 0.068 |
| 14 | 1-Penten-3-ol | A | 1160 | 17 | 0.092 ± 0.098 | 0.15 ± 0.15 |
| 15 | 3-Methylbutan-1-ol | A | 1210 | 4 | 0.91 ± 0.51 | 0.50 ± 0.44 |
| 16 | 3-Methyl-3-buten-1-ol | B | 1240 | 5 | 0.33 ± 0.15 | 0.23 ± 0.17 |
| 17 | 3-Methyl-2-buten-1-ol | B | 1335 | 1 | 0.29 ± 0.14 | 0.20 ± 0.13 |
| 18 | 1-Hexanol | A | 1357 | 13 | 0.20 ± 0.31 | 0.25 ± 0.16 |
| 19 | nonanal | A | 1397 | 18 | 0.13 ± 0.12 | 0.16 ± 0.17 |
| 20 | 1-Heptanol | A | 1453 | 19 | 0.037 ± 0.083 | 0.042 ± 0.053 |
| 21 | 2-Ethylhexanol | B,C | 1491 | 17 | 0.031 ± 0.020 | 0.031 ± 0.016 |
| 22 | benzaldehyde | A | 1506 | 11 | 0.88 ± 1.06 | 26.3 ± 10.7 |
| 23 | 2,3-butanediol | B | 1530 | n.d. | 0.016 ± 0.050 | 0.011 ± 0.012 |
| 24 | 1-octanol | B,C | 1558 | 13 | 0.060 ± 0.038 | 0.093 ± 0.045 |
| 25 | butyrolactone | B | 1633 | 14 | 0.017 ± 0.009 | 0.021 ± 0.008 |
| 26 | 1-Nonanol | B,C | 1655 | 15 | 0.028 ± 0.021 | 0.039 ±0.017 |
| 27 | methylsalicylate | B | 1757 | 20 | 0.018 ± 0.056 | 0.008 ± 0.008 |
| 28 | 2-Methoxyphenol | B,C | 1828 | 16 | 0.017 ± 0.035 | 0.034 ± 0.055 |
| 29 | phenylethyl alcohol | A | 1840 | 15 | 0.45 ± 0.31 | 1.29 ± 1.28 |
| 30 | benzyl alcohol | A | 1919 | 17 | 1.0 ± 0.6 | 1.0 ± 0.8 |

^a^: type of identification (A = by comparison of retention time and mass spectrum with those of reference compounds) or tentative identification (B = by comparison of the mass spectrum with those of the Wiley 6 and NIST libraries; C = by comparison of the linear retention index with those in the literature); ^b^: linear retention index; ^c^: intra-day precision of the analytical method, expressed as relative standard deviation; ^d^: mean concentration, expressed as μg equivalents of 4-Methyl-2-pentanol (IS)/g of almond; ^e^n.d.: not determined (not detected in the sample analyzed to assess the repeatability).

**Table S3.** Chromatographic areas obtained by analyzing ground almonds in suspension and by dry extraction (1 g sample, extraction at 40 °C during 30 min).

| **Compound** | **Chromatographic Area**  **(Mean ± Standard Deviation; *n* = 2)** | | | | | |
| --- | --- | --- | --- | --- | --- | --- |
|  | **Dry Extraction** | | | **Suspension** | | |
| acetaldehyde | 3079705 | ± | 153985 | 2767709 | ± | 55354 |
| ethylacetate | 2012879 | ± | 219569 | 2516099 | ± | 258316 |
| 2-Methylbutanal | 1721257 | ± | 213928 | 2458939 | ± | 245894 |
| 3-Methylbutanal | 3098263 | ± | 205691 | 4426090 | ± | 263706 |
| ethanol | 3821522 | ± | 391324 | 2547681 | ± | 407629 |
| pentamethyl heptane | 806223 | ± | 234785 | 1343705 | ± | 263803 |
| 2-Butanol | 1783557 | ± | 122301 | 2547938 | ± | 203835 |
| methyltridecane | 658402 | ± | 16084 | 823002 | ± | 22653 |
| 1-Propanol | 5876003 | ± | 265924 | 8770154 | ± | 302186 |
| hexanal | 12142263 | ± | 430098 | 7142508 | ± | 551408 |
| 2-Methylpropan-1-ol | 1844204 | ± | 196715 | 2458939 | ± | 245894 |
| 2-Pentanol | 5402200 | ± | 151847 | 7202934 | ± | 168719 |
| 1-Penten-3-ol | 2493332 | ± | 169070 | 3156117 | ± | 140892 |
| 3-Methylbutan-1-ol | 60932351 | ± | 1027508 | 92686875 | ± | 790391 |
| 3-Methyl-3-buten-1-ol | 9251992 | ± | 651220 | 13896053 | ± | 723577 |
| 3-Methyl-2-buten-1-ol | 9494802 | ± | 1073519 | 15338937 | ± | 1130020 |
| 1-Hexanol | 7242094 | ± | 709833 | 10165770 | ± | 887291 |
| nonanal | 2950821 | ± | 373003 | 3278690 | ± | 438827 |
| 1-Heptanol | 1516822 | ± | 84197 | 1896028 | ± | 85915 |
| 2-Ethylhexanol | 1061776 | ± | 45707 | 1327220 | ± | 60140 |
| benzaldehyde | 4422520 | ± | 467235 | 7149240 | ± | 502404 |
| 1-Octanol | 1385984 | ± | 204464 | 2321581 | ± | 252425 |
| butyrolactone | 168296 | ± | 25828 | 235874 | ± | 35381 |
| 1-Nonanol | 2014506 | ± | 109259 | 3224757 | ± | 125585 |
| 2-Methoxyphenol | 105252 | ± | 10510 | 131565 | ± | 11424 |
| phenylethyl alcohol | 7029454 | ± | 489177 | 11688484 | ± | 525997 |
| benzyl alcohol | 14770589 | ± | 2455400 | 25643384 | ± | 2822299 |
| Total area | 167087067 | ± | 10498179 | 237146267 |  | 11521965 |

**Table S4.** Results of the analysis of variance for the volatile compounds selected as possible biomarkers. Mean concentrations registered in different harvest years and geographical areas for heterozygous (*Sksk*) and homozygous/unknown (*SkSk, Sk--*) are reported, as well as significant differences (*p-*value ≤ 0.05).

|  | **Concentration (mg/kg)** | | | | | | |
| --- | --- | --- | --- | --- | --- | --- | --- |
| **Compound** | **Year** | | | **Geographical Area** | | | |
|  | **2012** | **2015** | ***p*-Value** | **1 ^a^** | **2 ^b^** | **3 ^c^** | ***p*-Value** |
| *Sksk* |  |  |  |  |  |  |  |
| 2-Methylpropanol | 0.12 | 0.01 | 0.006 | 0 | 0.1 | 0.2 | 0.000 |
| 3-Methylbutan-1-ol | 0.78 | 0.15 | 0.000 | 0.4 | 0.6 | 1.1 | 0.000 |
| 3-Methyl-3-buten-1-ol | 0.34 | 0.10 | 0.000 | 0.1 | 0.3 | 0.4 | 0.006 |
| 3-Methyl-2-buten-1-ol | 0.26 | 0.12 | 0.025 | 0.2 | 0.4 | 0.4 | 0.000 |
| benzaldehyde | 29.8 | 21.8 | 0.065 | 24.5 | 29.9 | 33.0 | 0.547 |
| benzyl alcohol | 1.49 | 1.02 | 0.357 | 1.2 | 1.8 | 1.5 | 0.879 |
| *SkSk, Sk--* |  |  |  |  |  |  |  |
| 2-Methylpropanol | 0,.19 | 0.03 | 0.002 | 0.08 | 0.30 | 0.21 | 0.000 |
| 3-Methylbutan-1-ol | 1.03 | 0.41 | 0.000 | 0.68 | 1.24 | 1.14 | 0.000 |
| 3-Methyl-3-buten-1-ol | 0.34 | 0.31 | 0.723 | 0.36 | 0.34 | 0.33 | 0.669 |
| 3-Methyl-2-buten-1-ol | 0.28 | 0.35 | 0.018 | 0.32 | 0.28 | 0.25 | 0.133 |
| benzaldehyde | 0.56 | 2.24 | 0.000 | 1.11 | 0.59 | 0.44 | 0.221 |
| benzyl alcohol | 0.43 | 0.39 | 0.018 | 0.48 | 0.47 | 0.32 | 0.133 |

^a^: Constantí (Tarragona, Spain); ^b^: Gandesa (Tarragona, Spain); ^c^: Les Borges Blanques (Lleida, Spain).

**Table S5.** Samples from homo- and heterozygous cultivars and selections, and their predicted values as the *SkSk* (homozygous) and *Sksk* (heterozygous) class of the PLS-DA model.

| **Year** | **Cultivar/Selection** | **Observed Class** | **Predicted Value as *SkSk* (Homozygous) Class** | **Predicted Value as *Sksk* (Heterozygous) Class** |
| --- | --- | --- | --- | --- |
| 2012 | Guara | *(Sksk)* heterozygous | −0.559 | 1.559 |
| 2012 | D. Largueta | *(Sksk)* heterozygous | −0.443 | 1.443 |
| 2012 | Guara | *(Sksk)* heterozygous | −0.422 | 1.422 |
| 2012 | D. Largueta | *(Sksk)* heterozygous | −0.366 | 1.366 |
| 2015 | Falsa Barese | *(Sksk)* heterozygous | −0.349 | 1.349 |
| 2012 | Guara | *(Sksk)* heterozygous | −0.320 | 1.320 |
| 2015 | Genco | *(Sksk)* heterozygous | −0.183 | 1.183 |
| 2012 | Guara | *(Sksk)* heterozygous | −0.149 | 1.149 |
| 2015 | Falsa Barese | *(Sksk)* heterozygous | −0.126 | 1.126 |
| 2012 | Guara | *(Sksk)* heterozygous | −0.124 | 1.124 |
| 2015 | Genco | *(Sksk)* heterozygous | −0.123 | 1.123 |
| 2012 | Guara | *(Sksk)* heterozygous | −0.099 | 1.099 |
| 2012 | D. Largueta | *(Sksk)* heterozygous | −0.074 | 1.074 |
| 2012 | Guara | *(Sksk)* heterozygous | −0.064 | 1.064 |
| 2012 | Marcona | *(Sksk)* heterozygous | −0.052 | 1.052 |
| 2012 | Guara | *(Sksk)* heterozygous | −0.026 | 1.026 |
| 2015 | Guara | *(Sksk)* heterozygous | −0.008 | 1.008 |
| 2012 | D. Largueta | *(Sksk)* heterozygous | 0.022 | 0.978 |
| 2012 | Marcona | *(Sksk)* heterozygous | 0.036 | 0.964 |
| 2012 | Guara | *(Sksk)* heterozygous | 0.046 | 0.954 |
| 2015 | Fltu18 | *(Sksk)* heterozygous | 0.106 | 0.894 |
| 2015 | IRTA-1 | *(Sksk)* heterozygous | 0.107 | 0.893 |
| 2015 | Guara | *(Sksk)* heterozygous | 0.142 | 0.858 |
| 2015 | FLTU18 | *(Sksk)* heterozygous | 0.143 | 0.857 |
| 2015 | Tuono | *(Sksk)* heterozygous | 0.145 | 0.855 |
| 2012 | Marta | *(Sksk)* heterozygous | 0.153 | 0.847 |
| 2015 | Tuono | *(Sksk)* heterozygous | 0.197 | 0.803 |
| 2015 | Gabaix | *(Sksk)* heterozygous | 0.197 | 0.803 |
| 2012 | Marta | *(Sksk)* heterozygous | 0.199 | 0.801 |
| 2015 | IRTA-1 | *(Sksk)* heterozygous | 0.214 | 0.786 |
| 2015 | FGTR13 | *(Sksk)* heterozygous | 0.214 | 0.786 |
| 2012 | Marta | *(Sksk)* heterozygous | 0.222 | 0.778 |
| 2012 | Marcona | *(Sksk)* heterozygous | 0.255 | 0.745 |
| 2012 | Guara | *(Sksk)* heterozygous | 0.266 | 0.734 |
| 2012 | Guara | *(Sksk)* heterozygous | 0.291 | 0.709 |
| 2015 | FGTR13 | *(Sksk)* heterozygous | 0.306 | 0.694 |
| 2012 | Marcona | *(Sksk)* heterozygous | 0.313 | 0.687 |
| 2012 | Marta | *(Sksk)* heterozygous | 0.323 | 0.677 |
| 2015 | Stelliete | *(Sksk)* heterozygous | 0.338 | 0.662 |
| 2015 | FGFP092 | *(Sksk)* heterozygous | 0.355 | 0.645 |
| 2015 | Gabaix | *(Sksk)* heterozygous | 0.411 | 0.589 |
| 2015 | Stelliete | *(Sksk)* heterozygous | 0.419 | 0.581 |
| 2012 | Marta | *(Sksk)* heterozygous | 0.444 | 0.556 |
| 2015 | Marcona | *(Sksk)* heterozygous | 0.473 | 0.527 |
| 2012 | Nonpareil | *(Sksk)* heterozygous | 0.478 | 0.522 |
| 2015 | Marcona | *(Sksk)* heterozygous | 0.493 | 0.507 |
| 2015 | FGFP092 | *(Sksk)* heterozygous | 0.527* | 0.473 |
| 2012 | Nonpareil | *(Sksk)* heterozygous | 0.553* | 0.447 |
| 2015 | Francolí | *(SkSk)* homozygous | 0.632 | 0.368 |
| 2012 | Nonpareil | *(Sksk)* heterozygous | 0.644* | 0.356 |
| 2015 | Francolí | *(SkSk)* homozygous | 0.652 | 0.348 |
| 2012 | Nonpareil | *(Sksk)* heterozygous | 0.681* | 0.319 |
| 2012 | Marinada | *(SkSk)* homozygous | 0.755 | 0.245 |
| 2012 | Vairo | *(SkSk)* homozygous | 0.760 | 0.240 |
| 2012 | Vairo | *(SkSk)* homozygous | 0.761 | 0.239 |
| 2012 | Glorieta | *(SkSk)* homozygous | 0.769 | 0.231 |
| 2012 | IRTA-9 | *(SkSk)* homozygous | 0.772 | 0.228 |
| 2012 | Glorieta | *(SkSk)* homozygous | 0.774 | 0.226 |
| 2012 | Lauranne | *(SkSk)* homozygous | 0.779 | 0.221 |
| 2015 | Masbovera | *(SkSk)* homozygous | 0.785 | 0.215 |
| 2012 | Lauranne | *(SkSk)* homozygous | 0.787 | 0.213 |
| 2012 | IRTA-9 | *(SkSk)* homozygous | 0.787 | 0.213 |
| 2012 | Francoli | *(SkSk)* homozygous | 0.788 | 0.212 |
| 2012 | Francoli | *(SkSk)* homozygous | 0.789 | 0.211 |
| 2015 | Marinada | *(SkSk)* homozygous | 0.792 | 0.208 |
| 2015 | Glorieta | *(SkSk)* homozygous | 0.799 | 0.201 |
| 2012 | Marinada | *(SkSk)* homozygous | 0.803 | 0.197 |
| 2015 | Ramillete | *(SkSk)* homozygous | 0.804 | 0.196 |
| 2012 | Francoli | *(SkSk)* homozygous | 0.809 | 0.191 |
| 2015 | Marinada | *(SkSk)* homozygous | 0.810 | 0.190 |
| 2012 | Marinada | *(SkSk)* homozygous | 0.811 | 0.189 |
| 2015 | Glorieta | *(SkSk)* homozygous | 0.815 | 0.185 |
| 2015 | Masbovera | *(SkSk)* homozygous | 0.817 | 0.183 |
| 2012 | Masbovera | *(SkSk)* homozygous | 0.822 | 0.178 |
| 2015 | Primorskiy | *(SkSk)* homozygous | 0.826 | 0.174 |
| 2012 | Francoli | *(SkSk)* homozygous | 0.830 | 0.170 |
| 2015 | Primorskiy | *(SkSk)* homozygous | 0.859 | 0.141 |
| 2012 | IRTA-12 | *(SkSk)* homozygous | 0.868 | 0.132 |
| 2012 | Francoli | *(SkSk)* homozygous | 0.872 | 0.128 |
| 2012 | Tarraco | *(SkSk)* homozygous | 0.872 | 0.128 |
| 2012 | Marinada | *(SkSk)* homozygous | 0.873 | 0.127 |
| 2012 | Tarraco | *(SkSk)* homozygous | 0.875 | 0.125 |
| 2015 | Vairo | *(SkSk)* homozygous | 0.881 | 0.119 |
| 2015 | Vairo | *(SkSk)* homozygous | 0.884 | 0.116 |
| 2012 | Tarraco | *(SkSk)* homozygous | 0.885 | 0.115 |
| 2012 | Tarraco | *(SkSk)* homozygous | 0.886 | 0.114 |
| 2012 | IRTA-9 | *(SkSk)* homozygous | 0.893 | 0.107 |
| 2012 | Marinada | *(SkSk)* homozygous | 0.902 | 0.098 |
| 2012 | IRTA-12 | *(SkSk)* homozygous | 0.902 | 0.098 |
| 2012 | IRTA-9 | *(SkSk)* homozygous | 0.902 | 0.098 |
| 2012 | Vairo | *(SkSk)* homozygous | 0.902 | 0.098 |
| 2012 | Tarraco | *(SkSk)* homozygous | 0.903 | 0.097 |
| 2012 | Marinada | *(SkSk)* homozygous | 0.906 | 0.094 |
| 2012 | IRTA-8 | *(SkSk)* homozygous | 0.907 | 0.093 |
| 2012 | Marinada | *(SkSk)* homozygous | 0.907 | 0.093 |
| 2012 | Marinada | *(SkSk)* homozygous | 0.907 | 0.093 |
| 2012 | Tarraco | *(SkSk)* homozygous | 0.908 | 0.092 |
| 2012 | IRTA-8 | *(SkSk)* homozygous | 0.909 | 0.091 |
| 2015 | Garbí | *(SkSk)* homozygous | 0.909 | 0.091 |
| 2012 | IRTA-10 | *(SkSk)* homozygous | 0.911 | 0.089 |
| 2012 | IRTA-12 | *(SkSk)* homozygous | 0.912 | 0.088 |
| 2012 | Tarraco | *(SkSk)* homozygous | 0.916 | 0.084 |
| 2012 | Francoli | *(SkSk)* homozygous | 0.921 | 0.079 |
| 2015 | Ramillete | *(SkSk)* homozygous | 0.923 | 0.077 |
| 2012 | Glorieta | *(SkSk)* homozygous | 0.925 | 0.075 |
| 2012 | Tarraco | *(SkSk)* homozygous | 0.927 | 0.073 |
| 2012 | Glorieta | *(SkSk)* homozygous | 0.927 | 0.073 |
| 2012 | Glorieta | *(SkSk)* homozygous | 0.928 | 0.072 |
| 2015 | 4-665 | *(SkSk)* homozygous | 0.928 | 0.072 |
| 2012 | Glorieta | *(SkSk)* homozygous | 0.929 | 0.071 |
| 2012 | IRTA-12 | *(SkSk)* homozygous | 0.930 | 0.070 |
| 2012 | Glorieta | *(SkSk)* homozygous | 0.931 | 0.069 |
| 2012 | Glorieta | *(SkSk)* homozygous | 0.933 | 0.067 |
| 2012 | Glorieta | *(SkSk)* homozygous | 0.933 | 0.067 |
| 2012 | Vairo | *(SkSk)* homozygous | 0.934 | 0.066 |
| 2012 | IRTA-12 | *(SkSk)* homozygous | 0.934 | 0.066 |
| 2012 | Marinada | *(SkSk)* homozygous | 0.934 | 0.066 |
| 2012 | Masbovera | *(SkSk)* homozygous | 0.934 | 0.066 |
| 2012 | Tarraco | *(SkSk)* homozygous | 0.936 | 0.064 |
| 2015 | IRTA-4 | *(SkSk)* homozygous | 0.936 | 0.064 |
| 2015 | Garbí | *(SkSk)* homozygous | 0.941 | 0.059 |
| 2012 | Tarraco | *(SkSk)* homozygous | 0.941 | 0.059 |
| 2012 | IRTA-10 | *(SkSk)* homozygous | 0.944 | 0.056 |
| 2012 | Vairo | *(SkSk)* homozygous | 0.945 | 0.055 |
| 2012 | Vairo | *(SkSk)* homozygous | 0.946 | 0.054 |
| 2012 | Marinada | *(SkSk)* homozygous | 0.950 | 0.050 |
| 2012 | Tarraco | *(SkSk)* homozygous | 0.950 | 0.050 |
| 2012 | Masbovera | *(SkSk)* homozygous | 0.951 | 0.049 |
| 2012 | Masbovera | *(SkSk)* homozygous | 0.951 | 0.049 |
| 2012 | IRTA-10 | *(SkSk)* homozygous | 0.954 | 0.046 |
| 2015 | Cristomorto | *(SkSk)* homozygous | 0.954 | 0.046 |
| 2012 | IRTA-10 | *(SkSk)* homozygous | 0.955 | 0.045 |
| 2012 | Masbovera | *(SkSk)* homozygous | 0.958 | 0.042 |
| 2012 | Tarraco | *(SkSk)* homozygous | 0.959 | 0.041 |
| 2012 | Vairo | *(SkSk)* homozygous | 0.959 | 0.041 |
| 2012 | Constantí | *(SkSk)* homozygous | 0.961 | 0.039 |
| 2012 | IRTA-12 | *(SkSk)* homozygous | 0.963 | 0.037 |
| 2012 | Vairo | *(SkSk)* homozygous | 0.964 | 0.036 |
| 2012 | Francoli | *(SkSk)* homozygous | 0.965 | 0.035 |
| 2012 | Constantí | *(SkSk)* homozygous | 0.966 | 0.034 |
| 2012 | Masbovera | *(SkSk)* homozygous | 0.967 | 0.033 |
| 2015 | Tarraco | *(SkSk)* homozygous | 0.968 | 0.032 |
| 2012 | Glorieta | *(SkSk)* homozygous | 0.971 | 0.029 |
| 2012 | Francoli | *(SkSk)* homozygous | 0.974 | 0.026 |
| 2012 | Ferragnes | *(SkSk)* homozygous | 0.976 | 0.024 |
| 2012 | Constantí | *(SkSk)* homozygous | 0.977 | 0.023 |
| 2012 | IRTA-8 | *(SkSk)* homozygous | 0.980 | 0.020 |
| 2012 | Marinada | *(SkSk)* homozygous | 0.980 | 0.020 |
| 2012 | Ferragnes | *(SkSk)* homozygous | 0.981 | 0.019 |
| 2012 | IRTA-8 | *(SkSk)* homozygous | 0.981 | 0.019 |
| 2012 | Constantí | *(SkSk)* homozygous | 0.982 | 0.018 |
| 2012 | Masbovera | *(SkSk)* homozygous | 0.982 | 0.018 |
| 2012 | Marinada | *(SkSk)* homozygous | 0.986 | 0.014 |
| 2012 | Constantí | *(SkSk)* homozygous | 0.989 | 0.011 |
| 2015 | IRTA-4 | *(SkSk)* homozygous | 0.990 | 0.010 |
| 2012 | IRTA-4 | *(SkSk)* homozygous | 0.992 | 0.008 |
| 2012 | Francoli | *(SkSk)* homozygous | 0.993 | 0.007 |
| 2012 | Constantí | *(SkSk)* homozygous | 0.994 | 0.006 |
| 2012 | Constantí | *(SkSk)* homozygous | 0.996 | 0.004 |
| 2012 | Constantí | *(SkSk)* homozygous | 0.997 | 0.003 |
| 2012 | Vairo | *(SkSk)* homozygous | 0.998 | 0.002 |
| 2012 | Francoli | *(SkSk)* homozygous | 0.998 | 0.002 |
| 2015 | Cristomorto | *(SkSk)* homozygous | 1.000 | 0.000 |
| 2012 | Vairo | *(SkSk)* homozygous | 1.003 | - 0.003 |
| 2012 | Constantí | *(SkSk)* homozygous | 1.005 | - 0.005 |
| 2012 | Masbovera | *(SkSk)* homozygous | 1.006 | - 0.006 |
| 2012 | Francoli | *(SkSk)* homozygous | 1.007 | - 0.007 |
| 2012 | Constantí | *(SkSk)* homozygous | 1.009 | - 0.009 |
| 2012 | Vairo | *(SkSk)* homozygous | 1.010 | - 0.010 |
| 2012 | Vairo | *(SkSk)* homozygous | 1.010 | - 0.010 |
| 2015 | Ferragnes | *(SkSk)* homozygous | 1.011 | - 0.011 |
| 2012 | Ferragnes | *(SkSk)* homozygous | 1.011 | - 0.011 |
| 2012 | IRTA-4 | *(SkSk)* homozygous | 1.013 | - 0.013 |
| 2012 | Glorieta | *(SkSk)* homozygous | 1.014 | - 0.014 |
| 2012 | Marinada | *(SkSk)* homozygous | 1.017 | - 0.017 |
| 2012 | Masbovera | *(SkSk)* homozygous | 1.021 | - 0.021 |
| 2012 | IRTA-4 | *(SkSk)* homozygous | 1.024 | - 0.024 |
| 2012 | IRTA-4 | *(SkSk)* homozygous | 1.024 | - 0.024 |
| 2012 | Glorieta | *(SkSk)* homozygous | 1.025 | - 0.025 |
| 2012 | Glorieta | *(SkSk)* homozygous | 1.025 | - 0.025 |
| 2012 | Masbovera | *(SkSk)* homozygous | 1.028 | - 0.028 |
| 2015 | 4-665 | *(SkSk)* homozygous | 1.029 | - 0.029 |
| 2012 | Ferragnes | *(SkSk)* homozygous | 1.031 | - 0.031 |
| 2015 | Tarraco | *(SkSk)* homozygous | 1.031 | - 0.031 |
| 2012 | Masbovera | *(SkSk)* homozygous | 1.033 | - 0.033 |
| 2012 | Constantí | *(SkSk)* homozygous | 1.043 | - 0.043 |
| 2012 | Constantí | *(SkSk)* homozygous | 1.045 | - 0.045 |
| 2012 | Belona | *(SkSk)* homozygous | 1.048 | - 0.048 |
| 2012 | Glorieta | *(SkSk)* homozygous | 1.058 | - 0.058 |
| 2012 | Belona | *(SkSk)* homozygous | 1.068 | - 0.068 |
| 2012 | Belona | *(SkSk)* homozygous | 1.070 | - 0.070 |
| 2012 | Lauranne | *(SkSk)* homozygous | 1.083 | - 0.083 |
| 2012 | Francoli | *(SkSk)* homozygous | 1.084 | - 0.084 |
| 2012 | Lauranne | *(SkSk)* homozygous | 1.086 | - 0.086 |
| 2015 | Ferragnes | *(SkSk)* homozygous | 1.089 | - 0.089 |
| 2012 | Francoli | *(SkSk)* homozygous | 1.095 | - 0.095 |
| 2012 | Constantí | *(SkSk)* homozygous | 1.101 | - 0.101 |
| 2012 | Constantí | *(SkSk)* homozygous | 1.102 | - 0.102 |
| 2012 | Belona | *(SkSk)* homozygous | 1.107 | - 0.107 |
| 2015 | IRTA-2 | *(SkSk)* homozygous | 1.108 | - 0.108 |
| 2012 | IRTA-4 | *(SkSk)* homozygous | 1.132 | - 0.132 |
| 2015 | IRTA-2 | *(SkSk)* homozygous | 1.144 | - 0.144 |
| 2015 | IRTA-3 | *(SkSk)* homozygous | 1.160 | - 0.160 |

classification threshold = 0.5; asterisk indicates uncorrected classifications.

**Table S6.** Samples from cultivars and selections with unknown genotype, and their predicted values as the *SkSk* (homozygous) class of the PLS-DA model.

| **Sample** | **Cultivar/Selection** | **Predicted Value as *SkSk* (Homozygous) Class** |
| --- | --- | --- |
| 1 | IRTA-7 | 1.026 |
| 2 | IRTA-7 | 1.003 |
| 3 | IRTA-7 | 1.017 |
| 4 | IRTA-7 | 1.040 |
| 5 | IRTA-7 | 0.998 |
| 6 | IRTA-7 | 0.976 |
| 7 | IRTA-7 | 0.936 |
| 8 | IRTA-7 | 0.957 |
| 9 | IRTA-7 | 1.027 |
| 10 | IRTA-7 | 1.071 |
| 11 | IRTA-7 | 1.111 |
| 12 | IRTA-7 | 0.976 |
| 13 | IRTA-7 | 0.986 |
| 14 | IRTA-7 | 1.186 |
| 15 | IRTA-7 | 1.209 |
| 16 | IRTA-7 | 0.945 |
| 17 | IRTA-7 | 0.935 |
| 18 | IRTA-11 | 0.779 |
| 19 | IRTA-11 | 0.800 |
| 20 | IRTA-11 | 1.026 |
| 21 | IRTA-11 | 1.092 |
| 22 | Cambra | 1.039 |
| 23 | Cambra | 0.950 |
| 24 | Cambra | 0.954 |
| 25 | Cambra | 0.975 |
| 26 | Felisia | 0.865 |
| 27 | Felisia | 0.892 |
| 28 | Felisia | 1.004 |
| 29 | Felisia | 0.998 |
| 30 | Soleta | 0.781 |
| 31 | Soleta | 0.774 |
| 32 | Soleta | 1.033 |
| 33 | Soleta | 1.017 |
| 34 | Soleta | 0.754 |
| 35 | Soleta | 0.754 |
| 36 | Soleta | 1.076 |
| 37 | Soleta | 1.083 |
| 38 | Soleta | 0.965 |
| 39 | Soleta | 0.958 |

classification threshold = 0.5.
